# Genome Assembly and Annotation for the Okinawan Green Marine Spoon Worm *Bonellia viridis* (Polychaeta: Bonelliidae)

**DOI:** 10.3390/ijms27125575

**Published:** 2026-06-20

**Authors:** Ezra M. Bailey, John Soghigian, Marcé D. Lorenzen, Ran Zhang, Masahiko Taniguchi, Jonathan S. Lindsey, Brian M. Wiegmann, Xiaohe Jin

**Affiliations:** 1 Department of Entomology and Plant Pathology, North Carolina State University, Raleigh, NC 27695, USA; embaile3@ncsu.edu (E.M.B.); mdlorenz@ncsu.edu (M.D.L.); bwiegman@ncsu.edu (B.M.W.); 2 Department of Entomology, Texas A&M University, College Station, TX 77843, USA; 3 Faculty of Veterinary Medicine, University of Calgary, Calgary, AB T2N 041, Canada; john.soghigian@ucalgary.ca; 4 Department of Chemistry, North Carolina State University, Raleigh, NC 27695, USA; rzhang0915@gmail.com (R.Z.); mtanigu@ncsu.edu (M.T.)

**Keywords:** Annelida, *Bonellia viridis*, bonellin, genome annotation, genome assembly, tetrapyrrole biosynthesis

## Abstract

*Bonellia viridis*, an echiuran polychaete that inhabits infralittoral rocky habitats around the Atlantic, Mediterranean, and Southeastern Pacific coastlines, exhibits environmentally mediated sexual dimorphism: planktonic larvae develop into dwarf males after exposure to bonellin, a green pigment produced by adult females. Bonellin is a chlorin with a structure consistent with derivation from uroporphyrinogen III, the last universal precursor of all known tetrapyrroles, yet its biosynthesis remains unknown. Here, the de novo genome assembly for a single adult female specimen of *B. viridis* isolated from Okinawa has been generated (via Illumina sequencing) and found to comprise 429.95 Mb across 95,859 contigs, with an N50 of 6505 bp, recovering 83.3% of near-universal metazoan BUSCO orthologs. Homologs of all canonical enzymes of the heme biosynthetic pathway (termed hem genes) were identified across the genome. The genomic resources establish a foundation for research into the biochemical basis of pigment production, chemically mediated sex determination, and the distinct biology of *B. viridis*.

## 1. Introduction

*Bonellia viridis* (Annelida: Polychaeta: Bonelliidae) is a “beautiful emerald green” [1] marine spoon worm belonging to the echiurans (Gk, *Echis*, “serpent-like” [1]), a small group of derived annelids characterized by the secondary loss of segmentation and distinctive morphological adaptations [2,3]. Within the broader diversity of polychaete annelids, echiurans occupy a unique evolutionary position and exhibit unusual life history strategies. Among them, *Bonellia* species are particularly notable for their extreme sexual dimorphism and specialized reproductive biology [1,4,5,6]. Indeed, *Bonellia* were noted as early as two centuries ago by Rolando [7] and are often featured in compendia of marine invertebrates [8]. Recent work highlights unexpected diversity among marine annelids, knowledge of which hinges on genomic data [9].

In the infralittoral species *B. viridis* [7], females are approximately 8–15 cm in length and possess an anterior proboscis that is highly elongated (up to 1.5 m in length) and bifurcated at the terminus, which is used for detritus feeding [6,8,10,11,12,13]. In contrast, males are dwarf (1–3 mm long), lack a proboscis, and typically live symbiotically within the female reproductive system, where they provide sperm in exchange for protection and nutrition, although exceptions have been reported [12,14].

A defining feature of *B. viridis* is its chemically mediated environmental sex determination. Adult females produce a physiologically active pigment, bonellin, which induces masculinization of planktonic larvae [15,16]. In addition to its developmental role, bonellin exhibits potent biological activity, including cytotoxic effects and possible defensive functions [6,17]. Although once thought to be derived from chlorophyll, bonellin (C_31_H_34_N_4_O_4_) is now known to be a unique chlorin structurally distinct from chlorophyll [18]. Despite considerable study of its biological effects, the biosynthetic origin of bonellin remains unknown. A major barrier to progress in this area has been the lack of genomic resources for *B. viridis*. Without a reference genome, it has been difficult to systematically identify candidate genes or investigate the molecular basis of pigment production. Generating a genome assembly for *B. viridis* is therefore an essential step for future studies of bonellin biosynthesis and for understanding the biology and evolution of this species.

We come to this problem as two teams with distinct perspectives. One team is intrigued by the possible use of novel enzymes for chemoenzymatic manipulation of tetrapyrrole macrocycles. One team is interested in evolutionary relationships, comparative genomics, and the elucidation of genetic mechanisms of interesting phenotypes. Here, we present the first de novo genome assembly for *B. viridis* (generated from Illumina sequencing data) and annotated using automated genome annotation pipelines. Using these genomic resources, the genes involved in tetrapyrrole biosynthesis have been identified as a first step toward understanding potential pathways yielding bonellin. Homologs of the complete canonical set of enzymes in the tetrapyrrole biosynthetic pathway were identified in the genome of *B. viridis*, including *hemA*, *hemB*, *hemC*, *hemD*, *hemE*, *hemF*, *hemY/G*, *hemH*, and *hemN*. The genomic resources presented here establish a foundation for future biochemical and functional studies aimed at elucidating the molecular mechanisms underlying bonellin production and chemically mediated sex determination in *B. viridis*.

## 2. Results

### 2.1. Genome Assembly and Feature Annotation—Overview

Tissue from the proboscis of a single adult female *B. viridis*, collected in Okinawa, was used to generate the results reported herein. The draft genome of *B. viridis* was assembled into 95,859 contigs with a total length of 429.9 Mbp. The assembly has an N50 of 6505 bp, with the largest contig reaching 52 kb, and an overall GC content of 41.6%, comparable to values reported for other marine invertebrate genomes [3,19,20]. Despite the substantial genome size, the assembly remains highly fragmented, with an L50 of 19,838 contigs. The large number of contigs and relatively short N50 likely reflect the challenges of assembling a repeat-rich eukaryotic genome using short-read sequencing data. Additional long-read sequencing and scaffolding strategies will be necessary to improve assembly contiguity and structural completeness. Thus, the present assembly is best regarded as a gene-content-oriented draft genome rather than a high-contiguity reference assembly suitable for clustering or structural genome organization analyses.

Assembly quality metrics and read-level characteristics were evaluated using FastQC and k-mer-based analyses, which indicated overall high sequencing quality and expected k-mer distributions (Tables S1–S3). GenomeScope analysis (k = 21) estimated a haploid genome size of ~364–366 Mb with moderate heterozygosity (~5.8–6.3%) and substantial repeat content (~183–185 Mb), consistent with the repeat-rich nature of the assembly (Table S2). Contamination assessment using BlobToolKit v4.5.0 [21] revealed a single dominant cluster consistent with *B. viridis*, with no evidence of substantial contamination from unrelated taxa (Figures S1 and S2). Repetitive sequences accounted for approximately 31.7% of the genome assembly based on RED-based repeat annotation. Genome completeness and annotation statistics are summarized in Table 1, including BUSCO assessment using the metazoa_odb10 dataset. Overall, 83.3% of metazoa BUSCO orthologs were complete and only 3.4% were missing completely, reflecting the relatively high completeness of the genome in terms of expected gene content.

Genome annotation using AUGUSTUS predicted 29,627 protein-coding genes across the assembly. BUSCO analysis of predicted protein sets indicated comparable completeness for models trained on *Caenorhabditis elegans* (37.4%) and *Drosophila melanogaster* (38.8%), whereas substantially lower completeness was observed for the *Schistosoma mansoni*-trained model (5.5%). For genome-scale visualization, coding sequences longer than 200 amino acids were selected to minimize fragmentation artifacts and highlight well-supported gene models. Under this threshold, 5198 coding sequences were located on the forward strand and 5205 on the reverse strand of the concatenated genome representation. The average predicted coding sequence length is 237 bp, and the average transcript length is 3805 bp. The unusually short average predicted CDS length likely reflects fragmented gene prediction in the current draft assembly rather than true biological coding sequence length. Accordingly, the annotation should be interpreted as a preliminary automated gene set. Coding sequences were distributed approximately evenly between forward and reverse strands, indicating no apparent strand bias across the genome. The unusually short average coding sequence length likely reflects the fragmented nature of the assembly and partial gene predictions rather than true biological gene sizes.

In addition to protein-coding genes, the genome contains a diverse set of noncoding RNAs, including 34 ribosomal RNA (rRNA) genes and 146 predicted transfer RNA (tRNA) genes. tRNA genes are distributed across multiple contigs, consistent with the fragmented nature of the assembly. rRNA genes are also detected at multiple loci rather than forming large contiguous clusters. Several localized regions of elevated GC content are observed, some of which correspond to rRNA loci and areas of increased coding density.

**Figure 1:**
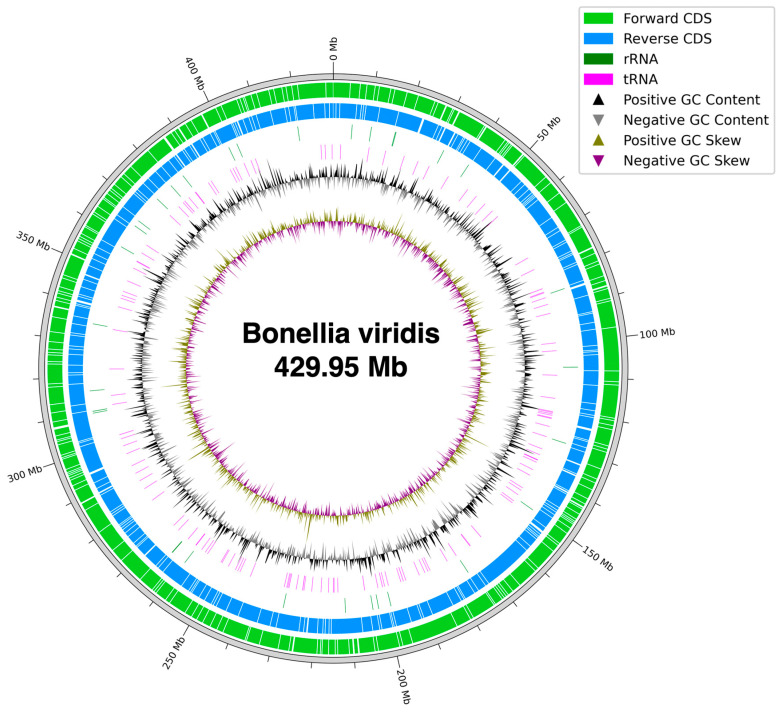
Circular genome representation of *B. viridis*. This pseudo-genome representation was generated by concatenating contigs for visualization only and does not represent chromosome structure or a circular genome. From outer to inner rings, the figure displays: outer tick marks indicating genomic position along the pseudo-genome; forward-strand coding sequence (CDS) density (green); reverse-strand CDS density (blue); noncoding RNA features, including predicted rRNA (dark green) and tRNA (magenta) genes; and GC content and GC skew tracks are shown as discrete markers along the genome. Only coding sequences longer than 200 amino acids were included in density tracks.

Figure 1 presents a circular representation of the genome of *B. viridis*, illustrating genome-wide patterns of GC content, GC skew, the distribution of tRNA and rRNA genes, and coding sequence density on both DNA strands. Forward- and reverse-strand coding densities are displayed separately to highlight strand symmetry and regional variation in gene content. Despite the fragmented nature of the assembly, this visualization provides a genome-wide overview of annotation features and facilitates exploration of gene distributions across distant genomic regions.

### 2.2. Phylogenetic Validation of the B. viridis Genome Assembly

**Figure 2:**
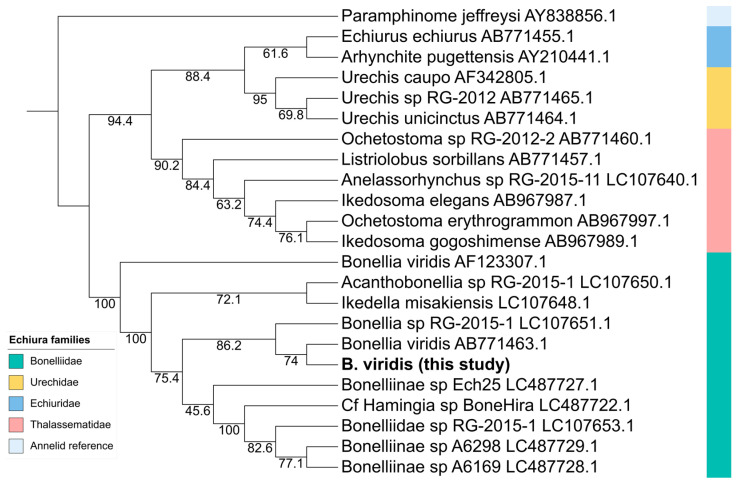
Maximum likelihood phylogenetic tree based on 18S rRNA gene sequences. The sequence obtained in this study is labeled as “***B. viridis* (this study)**”. Bootstrap support values are shown at internal nodes. Color strips indicate taxonomic assignments of included reference sequences.

Phylogenetic analysis based on the small-subunit ribosomal RNA (18S rRNA) gene was performed to support the taxonomic identity of the specimen used for genome assembly. The 18S rRNA sequence extracted from contig bviridis57874 clustered robustly with reference *B. viridis* sequences and closely related Bonelliidae sequences in the maximum-likelihood tree (Figure 2). The closest placement of the sequence from this study with a published *B. viridis* reference was supported by a bootstrap value of 74, and the broader Bonelliidae placement was supported by bootstrap values of 75.4–100 across relevant nodes [2,3]. Because this single-marker analysis was intended for specimen validation rather than reconstruction of deep echiuran or annelid relationships, deeper nodes were not interpreted further.

**Figure 3:**
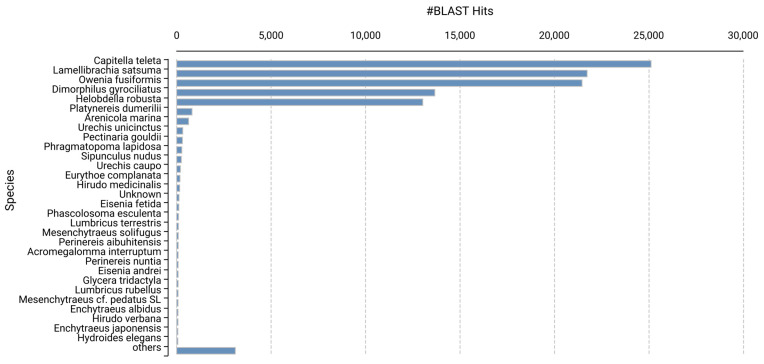
Transcript hits to possible species from all 30,625 sequences of the *B. viridis*. Illumina transcriptome assembly, with the majority BLAST v2.11.0 hits matching to *Capitella teleta*.

To provide additional context for gene content and taxonomic relationships, publicly available transcriptomic data from *B. viridis* were reassembled and functionally annotated. This independent dataset was not used for genome assembly or gene prediction but serves as a complementary resource for assessing sequence similarity and functional representation. Approximately 87.9 million raw paired-end Illumina reads were retrieved and reassembled, producing 30,625 transcript contigs with an average length of 648 bp, of which 4843 exceeded 1 kb in length (Tables S4 and S5). The transcriptome assembly was then imported into OmicsBox v3.4 (BioBam Bioinformatics) and annotations acquired using the Blast2GO v3 [22,23] module running the BlastX algorithm v2.11.0 [24] against NCBI’s non-redundant (nr) database of protein sequences from annelids. Of the 30,625 sequences, 15,761 yielded functional annotations, with the greatest number of matches (25,129) being proteins from the bristle worm, *Capitella teleta* (Figure 3). Gene ontology classification further indicated that the largest number of annotated transcripts were associated with cellular processes, followed by biological regulation (Figure S3).

### 2.3. Analysis for Bonellin

To examine the *B. viridis* specimens for the presence of bonellin, crude extracts were prepared and analyzed by absorption spectroscopic and mass spectrometric methods. The absorption spectrum recorded in toluene at room temperature exhibited a characteristic Soret (B) band at 393 nm and a Q_y_ band at 642 nm, consistent with chlorin-type tetrapyrrole chromophores and in agreement with reported values for bonellin dimethyl ester [25].

Mass spectrometric analysis revealed a peak at *m*/*z* 527.26445, consistent with the theoretical *m*/*z* 527.26528 for the [M + H]^+^ species wherein M = C_31_H_34_N_4_O_4_, the molecular formula of bonellin. The mass accuracy (ΔM = −1.574 ppm) was well within the accepted standard of ±5 ppm for correct compositional assignment.

Together, these absorption and mass data support the presence of bonellin in the analyzed specimens and support subsequent genomic investigation of pathways potentially involved in tetrapyrrole biosynthesis.

### 2.4. Identification of Tetrapyrrole Biosynthesis Genes in B. viridis

Homolog-based searches identified candidate loci corresponding to enzymes of the conserved heme/tetrapyrrole biosynthetic pathway in *B. viridis*, including *hemA*, *hemB*, *hemC*, *hemD*, *hemE*, *hemF*, *hemY/hemG*, *hemH*, and *hemN*. Multiple homologous loci were detected for several enzymes (Table 2). For each canonical enzymatic step, at least one candidate homologous region was recovered; however, several gene models remain partial in the current draft assembly and preliminary annotation. Among these, a subset corresponded to complete coding sequences, whereas others represented truncated or fragmented gene models. These partial copies likely reflect incomplete gene predictions or remnants of duplicated loci within the fragmented genome assembly.

For the early steps of the pathway, a full-length *hemA* gene encoding 5-aminolevulinate synthase (ALAS) was identified together with two truncated homologs. The *hemB*, *hemC*, and *hemD* genes were also detected, including one complete copy of each enzyme required for the conversion of δ-aminolevulinic acid to uroporphyrinogen III (Uro’gen III). Multiple homologs were identified for *hemE*, which encodes uroporphyrinogen decarboxylase (UROD), including one full-length gene and several truncated or fragmented copies. Domain analyses confirmed that these sequences contain conserved UROD-like motifs.

Genes encoding the downstream oxidation steps of the pathway were also present. Several homologs of *hemF* were detected, encoding oxygen-dependent coproporphyrinogen III oxidase. These homologs include one full-length copy and multiple truncated or fragmented candidates. Three homologs of *hemY*/*hemG*, encoding protoporphyrinogen oxidase, were identified, including one full-length gene and additional truncated or fragmented sequences. A single radical *S*-adenosylmethionine (SAM)–dependent coproporphyrinogen oxidase (HemN) homolog was also identified. Finally, two candidate *hemH* genes encoding ferrochelatase were detected, including one full-length copy and one fragmented sequence, completing the enzymatic steps required for heme biosynthesis.

**Figure 4:**
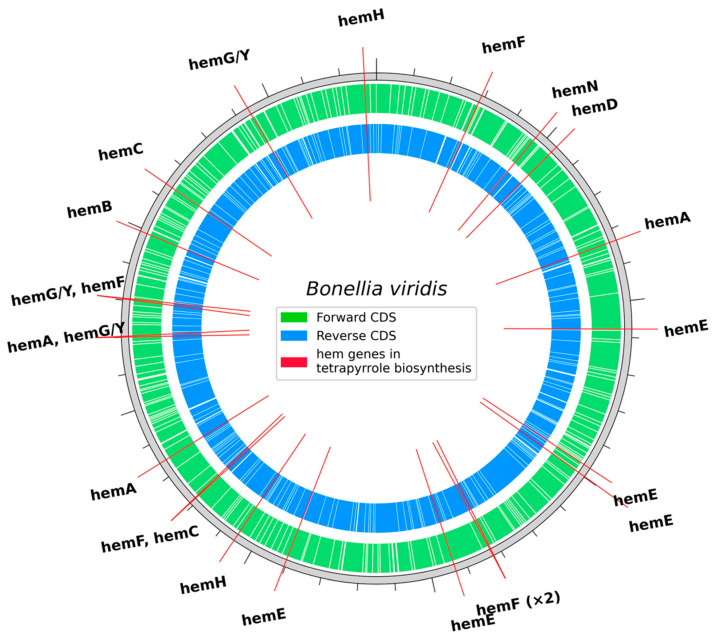
Locations of candidate hem homologs in the concatenated draft assembly of *B. viridis*. Candidate loci (highlighted with red blocks) are shown on the pseudo-genome representation for visualization only.

Candidate hem genes are distributed across multiple contigs in the current assembly (Figure 4). Given the low contiguity of the assembly, however, this observation cannot be used to determine whether these loci are physically linked, dispersed, or clustered in the native genome. The copy numbers listed here should be regarded as preliminary counts of homologous regions of recovered candidates, not definitive biological gene copy numbers.

## 3. Discussion

### 3.1. Molecular Structural Interpretation and Implications for Bonellin Biosynthesis

The identification of a complete set of tetrapyrrole biosynthesis genes provides a genomic framework for beginning to examine the biosynthetic origin of bonellin. To further assess this relationship, the molecular structure of bonellin was examined in the context of tetrapyrrole metabolism.

**Figure 5:**
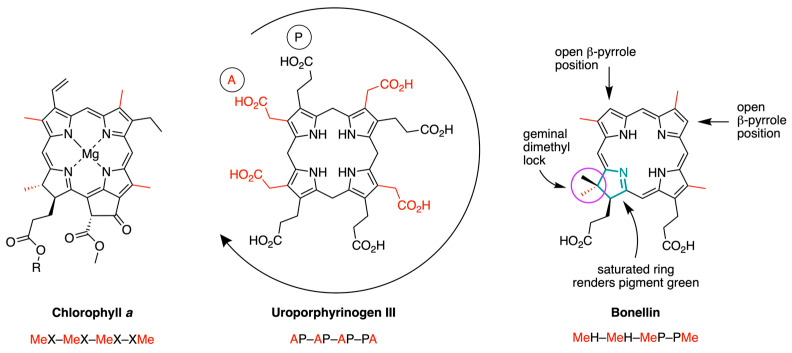
Structures of chlorophyll *a*, uroporphyrinogen III, and bonellin. Each substituent shown in red in chlorophyll *a* and in bonellin is likely derived from an acetic acid substituent (also shown in red) of uroporphyrinogen III. The pattern of substituents is a telltale signature of biosynthetic origin, is summarized beneath the name (beginning in upper left), and is obtained upon proceeding clockwise about the macrocycle, where A = acetic acid, P = propionic acid, Me = methyl, H = hydrogen, and X indicates one of several substituents. Bonellin has distinctive structural features (shown by arrows at right)—including two open β-pyrrole positions, a saturated ring (displayed with green bonds), and a geminal dimethyl group—which together set this macrocycle apart from all other known native tetrapyrroles.

The structure of bonellin [26,27] is shown in Figure 5. The structures of chlorophyll *a* and uroporphyrinogen III are also displayed. Uroporphyrinogen III is the most biosynthetically advanced universal precursor of all tetrapyrroles, standing at the nexus where branching occurs, leading to heme and chlorophylls, or to cobalamin, sirohemes, and F_430_ [28,29]. Understanding the evolutionary origin of tetrapyrrole biosynthesis and associated proteins remains a very active area of research; indeed, some bilin pigments found in cyanobacteria are suggested to have been acquired from non-photosynthetic bacteria [30].

Uroporphyrinogen III has a characteristic pattern of substituents arrayed about the perimeter of the macrocycle: whereas each pyrrole contains one acetic acid (A) and one propionic acid (P) substituent, the pattern upon circumambulating the ring (clockwise from upper left) is AP-AP-AP-PA; in other words, the orientation of the final ring is inverted (Figure 5, center structure). Subsequent biosynthetic manipulations alter the A and P groups, but the core pattern of one reversed pyrrole ring remains and is evident in all downstream tetrapyrrole macrocycles including heme, chlorophylls, cobalamin and sirohemes [31]. The same pattern is also evident in bonellin, offering a compelling molecular argument for biosynthetic origin from uroporphyrinogen III.

Bonellin also contains a reduced (saturated) ring, affording a chlorin chromophore. Although chlorin-based systems are often associated with photosynthetic or photosymbiotic functions, bonellin is not involved in photosynthesis, and *B. viridis* does not represent a “plant-like animal” as described in other animal lineages [32]. Chlorins in nature are hardly rare, as chlorophyll *a* is a chlorin, but the two chlorins differ in the substituent pattern in the saturated ring: a gem-dimethyl substituent in bonellin versus a *trans*-dialkyl substituent in chlorophyll *a* [25]. The gem-dimethyl group has proved of great value in the synthesis of non-native chlorins [33] and bacteriochlorins [34]. On the other hand, bonellin is in rare company among the large family of native tetrapyrroles in the presence of two open β-pyrrole positions (Figure 5, right structure), presumably due to the loss of two propionic acid substituents. Members of the tetrapyrrole family known to be derived by loss of two propionic acid substituents include the exotic macrocycles tolyporphins [35,36], found in a filamentous cyanobacterium; heme *d*_1_ [29], present in denitrifying bacteria; and corralistins [37] and isabellins [38], found in marine sponges. The limited studies to date indicate more than one mechanism for removal of the propionic acid groups [39].

Taken together, the genomic data identify candidate homologs of the conserved heme/tetrapyrrole pathway, providing a preliminary gene-content framework for future studies. However, the specific enzymatic steps responsible for the distinct structural modifications of bonellin, including side-chain removal and ring reduction, remain unresolved. No dedicated biosynthetic gene clusters (as found for tolyporphin biosynthesis [40]) or clearly identifiable pathway-specific enzymes were detected, suggesting that bonellin biosynthesis may proceed through modification of canonical tetrapyrrole intermediates by general metabolic enzymes, consistent with the dispersed genomic organization observed.

### 3.2. Limitations of the Current Study

Several limitations of this study should be acknowledged.

First, the genome assembly of *B. viridis* remains highly fragmented, which likely contributes to incomplete gene models and the large number of short coding sequences. Although the current assembly was sufficient for identifying candidate genes involved in tetrapyrrole metabolism and bonellin biosynthesis, improved assemblies generated with higher sequencing coverage and long-read technologies would greatly enhance gene prediction accuracy and genome continuity. Also, the GenomeScope results indicate high heterozygosity within the sequenced specimen, which may have contributed to assembly fragmentation and duplicated BUSCOs.

Second, gene annotation relied on parameter sets from *C. elegans* and *D. melanogaster* because the closer relative *S. mansoni* produced substantially lower completeness scores in BUSCO evaluations (5.5% compared to ~37–39% for the other models), highlighting the current lack of suitable training models for this lineage. In addition, biosynthetic genes in eukaryotes are often dispersed rather than organized in many bacterial-like gene clusters. While the present draft genome does not resolve the molecular mechanism of bonellin-mediated sex determination, the data obtained should provide a preliminary resource for future work.

Third, a key concern at the outset of this work was whether the origin of bonellin in *B. viridis* stemmed from biosynthesis by a symbiotic microorganism rather than by *B. viridis* itself. The absence of substantial contamination from unrelated taxa at first glance suggests an endogenous biosynthetic pathway for bonellin; however, note that only the tissue from the proboscis was used for DNA analysis. The occurrence of bonellin biosynthesis by a symbiotic organism located in other tissues, followed by dissemination of bonellin within *B. viridis*, cannot be ruled out. A dietary source also cannot be excluded.

Last, a deeper understanding of the phylogenetic position of *B. viridis* within Echiura and Annelida will require future collection efforts and additional sequencing. Specimens from Okinawa, the Mediterranean region, and other localities would likely provide valuable data for evaluating geographic variation, species boundaries, genome evolution, and the distinctive biological features of these unusual green worms.

## 4. Materials and Methods

### 4.1. Organism Collection and Identification

**Figure 6:**
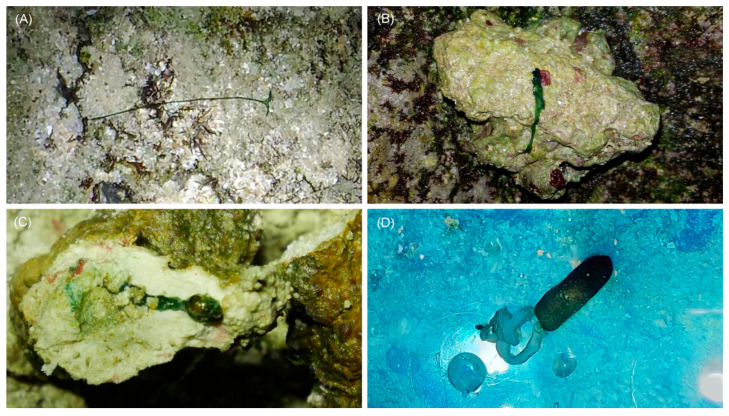
Four specimens of *B. viridis* in/on infralittoral rocks at Odo beach in Okinawa, displayed here with photographic images as seen by the unaided eye: (**A**) a specimen with proboscis (length >30 cm) extended in foraging; (**B**) a specimen with proboscis length of ~5 cm; (**C**) a collected specimen with body ~1 cm in length; (**D**) a collected specimen with body ~1.5 cm in length. Reprinted/adapted with permission from Ref. [41]. Copyright 2022, SPIE.

A total of 28 samples of *B. viridis* were collected from Okinawa, Japan, during three visits by one of us (MT) coincident with low tides. The specimens were identified as *B. viridis* on the basis of habitat, morphology, lifestyle, feeding behavior, color, and presence of bonellin. Specimens of *B. viridis* observed in situ were sensitive to light and vibrations, and no proboscides were observed as extended and foraging in the daytime. The samples were collected in the mid-intertidal zone at Odo shore, Itoman city, Okinawa prefecture, Japan [26°09′ N, 127°71′ E] at approximately 300 m distance from the shore at midnight on days when the tides were very low: 6–12 March 2016; 22–27 December 2018; and 18–25 January 2019 [41]. The main body trunk of *B. viridis* (Figure 6) typically sits inside a burrow cavity with the proboscis extended at night for feeding. Individuals can be obtained in small tidal pools (approximately 2 m × 2 m) of a depth reachable by hand (approximately 0.5 m) from small soft calcareous or coral rocks. In these pools, adult females were observed with the proboscis (typically less than 15 cm) protruding from the burrow (Figure 6). Rocks were removed from the tidal pool, then broken to reveal the burrow entrance and specimen. In the collected individuals, the main body trunk was 1–2 cm and the observed length of the proboscis was 10–15 cm. Ten specimens were collected and immediately stored in the preservative buffer RNALater (Thermo Fisher Scientific, Waltham, MA, USA). Ultimately, one such specimen was used within days of the 2019 collection to generate the genomic data described herein.

### 4.2. Extraction and Analysis for Bonellin

Following a procedure for extraction of tolyporphins from the filamentous non-axenic cyanobacterial culture HT-58-2 [42], one specimen of *B. viridis* (from 2019, and stored in RNALater) in its entirety was homogenized in an ice-cold mixture of dichloromethane/isopropanol (1:1). Filtration of the homogenate by centrifugation afforded a green filtrate, which was concentrated to dryness via a stream of nitrogen.

A portion of the crude solid was dissolved in toluene for absorption spectroscopic analysis, following conditions comparable to those reported for bonellin analogues [25], while a separate portion was dissolved in a mixture of acetonitrile/water (1:1) for mass spectrometric analysis. The latter solution was analyzed on a high-resolution mass spectrometer (Thermo Fisher Scientific Exactive Plus MS, Waltham, MA, USA, a benchtop full-scan Orbitrap mass spectrometer with resolution 70,000) using a heated electrospray ionization source (capillary temperature 350 °C, heater temperature 300 °C, spray voltage 3.5 kV). The sample was introduced via syringe injection (flow rate 15 µL/min) and analyzed in positive ion mode.

### 4.3. DNA Extraction, Library Preparation, and Sequencing

Genomic DNA for Illumina sequencing was taken exclusively from the proboscis tissue of a single adult female *B. viridis* (stored in RNALater) and extracted using the MagAttract HWM DNA Kit (Qiagen, Venlo, The Netherlands). The proboscis was used exclusively to avoid contamination from DNA of any males inside the body. DNA was quantified using the Qubit 2.0 DNA kit according to the manufacturer’s instructions (Invitrogen, Waltham, MA, USA). Genetic material for whole-genome sequencing was submitted to the Genomic Sciences Laboratory (GSL) at North Carolina State University (NCSU) for library preparation and sequencing. For Illumina sequencing, libraries were prepared according to the manufacturer’s protocol using the NEBNext Ultra II DNA Library Prep Kit (New England Biolabs, Ipswich, MA, USA). The sequencing of the final library was performed with the Illumina NovaSeq 6000 platform using 150 bp paired-end reads.

### 4.4. Genome Assembly

Raw FASTQ reads were quality checked using KMC v2.2.0 [43], GenomeScope 2.0 [44], and FastQC v0.12.0 [45]. Illumina sequencing generated 260,540,372 reads, corresponding to 39.3 Gbp of raw sequence data, with a read length of 151 bp and GC content of 42%. No sequences were flagged as poor quality by FastQC. Based on the final assembly size of 429.95 Mb, the raw sequencing depth was approximately 91×. KMC analysis was performed with k = 27, and GenomeScope 2.0 analysis was performed with k = 21 in diploid mode. Reads were trimmed with fastp v1.3.3 [46] and assembled using GATB-minia v0.0.102 with default parameters [47]. An independently assembled transcriptome dataset (SRA: SRR2017645) was analyzed separately (see Supplementary Materials) but was not used for genome assembly or gene prediction. The relative completeness of the genome assembly was evaluated by comparison with the universal single-copy orthologs from the Metazoa databases obtained from OrthoDB v10 [48] using BUSCO v5.8.0 [49] (E-value cutoff 0.001).

### 4.5. Genome Annotation and Repeat Identification

Gene prediction was performed on the assembled genome of *B. viridis* using the MOSGA v1 genome annotation pipeline [50,51]. Prior to gene prediction, the genome assembly was soft masked to reduce the influence of repetitive sequences on gene model prediction. Repeat masking and identification were conducted using RED with a minimum repeat size threshold of 100 bp [52], and repeat annotations were retained in GFF format for downstream analyses. Non-coding RNA genes were also annotated as part of the genome characterization process. Transfer RNA genes were identified using tRNAscan-SE v2.0 with a minimum score threshold of 70 [53]. Ribosomal RNA genes were predicted using Barrnap v0.9 with default parameters to detect conserved rRNA gene regions across the genome assembly (https://github.com/tseemann/barrnap, accessed on 28 May 2026). The AUGUSTUS gene prediction module (v3.4.0) was applied with multiple metazoan training parameter sets to improve gene model recovery in this fragmented genome assembly [54,55]. Three training species models were selected for the genome annotation: *C. elegans*, *D. melanogaster*, and *S. mansoni*. Annotation completeness was assessed using BUSCO with the metazoan reference dataset (n = 954) [56]. Because no close annelid or echiuran AUGUSTUS training model was available in the annotation implementation used here, these gene predictions should be considered preliminary.

### 4.6. Phylogenetic Analysis Based on 18S rRNA

To support the taxonomic assignment of the specimen used for genome assembly, phylogenetic analysis was performed using the small-subunit ribosomal RNA (18S rRNA) gene. Ribosomal RNA features were identified in the genome assembly using Barrnap. Two 18S-like rRNA features were initially recovered. The sequence from contig bviridis57874 showed highest BLASTN similarity to published *Bonellia viridis* 18S rRNA sequences and was used for taxonomic validation. A second rRNA/ITS-like feature showed highest BLAST similarity to a fungal ribosomal DNA sequence (*Verticillium dahliae*) and was not used for specimen validation. Because the purpose of this analysis was to support assignment of the assembled target organism, subsequent phylogenetic analysis was restricted to the Bonellia-like 18S rRNA sequence. Sequences were aligned by MAFFT v7 [57] with default parameters. Phylogenetic relationships were inferred using the maximum-likelihood method implemented in IQ-TREE v2.4.0 [58]. Branch support was assessed using bootstrap analysis with 1000 replicates.

### 4.7. Identification of Tetrapyrrole Biosynthesis Genes

Genes involved in tetrapyrrole and heme biosynthesis were identified using homology-based searches. Protein sequences of known enzymes from representative metazoans [59], including *Capitella teleta*, *Helobdella robusta*, and *Homo sapiens*, were used as queries for TBLASTN searches (https://blast.ncbi.nlm.nih.gov, accessed on 28 May 2026) against the *B. viridis* genome assembly. Searches were performed using an E-value threshold of 1 × 10^−5^, and candidate loci were retained when the alignment coverage exceeded 30–40% of the query length. Genomic regions identified by TBLASTN were compared with predicted gene models from the MOSGA/AUGUSTUS annotation to determine candidate coding sequences [60]. Predicted protein sequences were extracted from the annotation using AGAT utilities and manually translated when necessary to resolve strand orientation and premature stop codons caused by incomplete gene models [61].

### 4.8. Computational Validation of Candidate Genes

Candidate protein sequences were validated using BLASTP searches (https://blast.ncbi.nlm.nih.gov, accessed on 28 May 2026) against the NCBI nonredundant (nr) protein database. Hits with significant similarity to known tetrapyrrole biosynthesis enzymes were retained. Protein domain architecture was further examined using InterProScan (https://www.ebi.ac.uk/interpro, accessed on 28 May 2026) to confirm the presence of conserved catalytic domains characteristic of heme pathway enzymes [62]. Gene models were classified into three categories based on protein completeness relative to homologous sequences in other metazoans:

1. Full-length: predicted proteins with near-complete length and conserved catalytic domains.
2. Truncated: sequences missing substantial N- or C-terminal regions but still containing identifiable domains.
3. Fragmented: short partial sequences lacking complete domain architecture, typically resulting from assembly fragmentation.

These categories refer to the current predicted models relative to known homologs and should not be interpreted as definitive biological gene states. Genomic coordinates of validated loci were extracted from the corresponding GFF annotation files and manually curated to produce a comprehensive list of tetrapyrrole biosynthesis genes.

## 5. Conclusions

The emerald green spoon worm *B. viridis* has provoked a sense of wonder for more than two centuries [7]. In an effort to understand the biosynthetic origin of the green pigment, bonellin, a specimen from Okinawa has been used to generate a genome with high completeness. The present 430 Mbp genome assembly comprising 95.9k contigs was recovered as 83.3% complete using BUSCOs. Bonellin displays structural features consistent with derivation from uroporphyrinogen III, the last universal macrocyclic precursor to all naturally occurring tetrapyrroles. The complete canonical set of enzymes was identified for the heme biosynthetic pathway, including *hemA*, *hemB*, *hemC*, *hemD*, *hemE*, *hemF*, *hemY/G*, *hemH*, and *hemN*, indicating that *B. viridis* possesses the conserved metabolic framework required for tetrapyrrole production. This draft assembly should provide a useful preliminary genomic resource for *B. viridis*. Future work with improved long-read assemblies and transcript-supported annotation will be needed to resolve gene structure and identify the enzymes responsible for bonellin-specific modifications. Looking further, improved genome assemblies and transcript-supported annotations may enable searches for candidate receptors, transporters, neuroendocrine signaling components, and developmental regulators in larval responses to bonellin. In short, extensive studies are required to understand the biochemistry, chemistry, enzymology, and physiology of the long-known yet still-mysterious emerald-green annelid *B. viridis*.

## Figures and Tables

**Table 1:** Summary statistics of the *B. viridis* genome assembly and annotation.

Summary of Genome Assembly	
Total number of contigs	95,859
Total assembly length (bp)	429,954,275
Average contig length (bp)	4484
Percentage of Ns	1.50%
Shortest contig (bp)	1000
Longest contig (bp)	52,029
N50 (bp)	6505
L50 (contigs)	19,838
GC content (%)	41.57
Repeat content (%)	31.7% (RED)
BUSCO assessment (metazoa_odb10, n = 954)	
Complete BUSCOs (C)	83.3%
Complete single-copy (S)	52.1%
Complete duplicated (D)	31.2%
Fragmented BUSCOs (F)	13.3%
Missing BUSCOs (M)	3.4%

**Table 2:** Genomic loci and characteristics of tetrapyrrole biosynthesis genes in *B. viridis*.

Gene	Enzyme	Copy	Contig	Start	End	Strand	Length	Status
*hemA*	5-Aminolevulinate synthase (ALAS)	1	bviridis18240	5027	12,635	−	590	Full
		2	bviridis63199	2356	7080	+	344	Fragment
		3	bviridis70912	316	3418	+	181	Fragment
*hemB*	δ-Aminolevulinic acid dehydratase (ALAD)	1	bviridis77720	8727	12,705	−	268	Full-length
*hemC*	Porphobilinogen deaminase	1	bviridis60521	396	1983	+	161	Fragment
		2	bviridis80983	2746	6541	+	357	Full-length
*hemD*	Uroporphyrinogen-III synthase (UROS)	1	bviridis11783	2196	13,910	+	282	Full-length
*hemE*	Uroporphyrinogen decarboxylase (UROD)	1	bviridis23825	1264	4691	−	192	Fragment
		2	bviridis32729	1953	6845	−	329	Truncated
		3	bviridis33284	1206	6226	+	373	Full-length
		4	bviridis42902	3776	5378	+	186	Fragment
		5	bviridis53552	299	2320	+	193	Fragment
*hemF*	Oxygen-dependent coproporphyrinogen-III oxidase	1	bviridis6458	992	7590	+	341	Full-length
		2	bviridis40338	1567	5465	+	272	Truncated
		3	bviridis40876	1196	4357	+	199	Fragment
		4	Bviridis60095	996	1840	+	107	Fragment
		5	bviridis73707	357	3205	−	236	Truncated
*hemY*/ *hemG*	Protoporphyrinogen/copro-porphyrinogen III oxidase	1	bviridis71453	5186	9321	+	407	Truncated
		2	bviridis73189	7798	12,165	−	499	Full-length
		3	bviridis87735	206	886	+	112	Fragment
*hemH*	Protoporphyrin/copropor-phyrin ferrochelatase	1	bviridis56911	616	9476	+	400	Full-length
		2	bviridis95120	1066	1787	+	154	Fragment
*hemN*	Oxygen-independent coproporphyrinogen-III oxidase	1	bviridis10518	1848	6984	−	407	Full-length

## Data Availability

The original contributions presented in this study are included in the article/Supplementary Materials. All raw sequencing data have been deposited in the National Center for Biotechnology Information (NCBI Sequence Read Archive SRR39222284). Metadata concerning the biosamples and genomic sequences are available from NCBI (PRJNA1479034, SAMN60993020). Further inquiries can be directed to the corresponding authors.

## Supplementary Materials

The following supporting information can be downloaded at: https://www.mdpi.com/article/10.3390/ijms27125575/s1.
