# Supplementary material for: Genome Assembly and Annotation for the Okinawan Green Marine Spoon Worm Bonellia viridis (Polychaeta: Bonelliidae)
Source: Int J Mol Sci. 2026 Jun 20;27(12):5575. doi: 10.3390/ijms27125575 (PMC13299783; doi:10.3390/ijms27125575)
Supplement: Supplementary file 1 [file ijms-27-05575-s001.zip › ijms-4311873-supplementary.pdf]

Supplementary Material for:

Genome Assembly and Annotation for the Okinawan Green Marine Spoon Worm *Bonellia viridis* (Polychaeta: Bonelliidae)

Illumina Sequence Quality

The program KMC 2.2.0 [43] was run with the "fastq" file of all concatenated genomic sequences using the "*kmc*" command with the recommended parameters of "*-k27 -ci1 -cs500000*" and recovered a total of 260540372 reads (Table S1).

Table S1. KMC k-mer counter results of reads and k-mers using a k-mer of 27.

No. of k-mers below min. threshold	0
No. of k-mers above max. threshold	0
No. of unique k-mers	10358481041
No. of unique counted k-mers	10358481041
Total no. of k-mers	32564249259
Total no. of reads	260540372
Total no. of super-k-mers	3345515438

The histogram file was then created using the "*kmc_tools transform*" command with the parameter of "*-cx500000*". Results were duplicated successfully on a separate machine and KMC workflow. GenomeScope 2.0 [44] was run locally according to the developer's manual with "*21*" as the k-mer size, widely recommended for limiting the effects of heterozygosity and sequencing error, and with "*2*" as the ploidy (Table S2).

Table S2. GenomeScope 2.0 results with a k-mer of 21.

**Property**	**Min**	**Max**
Homozygous (aa)	93.7199%	94.1755%
Heterozygous (ab)	5.82447%	6.28011%
Genome Haploid Length	364,449,099 bp	366,115,238 bp
Genome Repeat Length	183,741,151 bp	184,581,154 bp
Genome Unique Length	180,707,948 bp	181,534,084 bp
Model Fit	62.3069%	95.1695%
Read Error Rate	1.6651%	1.6651%

The program Fastqc 0.12.0 [45] was run to check sequencing error rate (Table S3). Almost all measures received an "*excellent*" score. The only measure with a "*good*" score was the "*Per Base Sequence Content*". This result is known to be associated with Illumina amplicon sequencing. Sequence content across all bases equalized at about 20 bases per 151 bases in each sequence.

Table S3. Fastqc results of Illumina paired-end sequences.

File type	Conventional base calls
Encoding	Sanger / Illumina 1.9
Total Sequences	260540372
Total Bases	39.3 Gbp
Sequences flagged as poor quality	0
Sequence length	151
% GC	42

The program BlobToolKit [21] was first used to assess clustering of sequences and recovered a single cluster with a square-binned blob plot (Figure S1). Next, a cumulative assembly span plot was used to again assess clustering of sequences and visualize the cumulative length and contiguity of the assembly by record hit number (Figure S2). No evidence of contamination was exhibited, such as recovering more than a single cluster or significant record hits of an unrelated taxon.

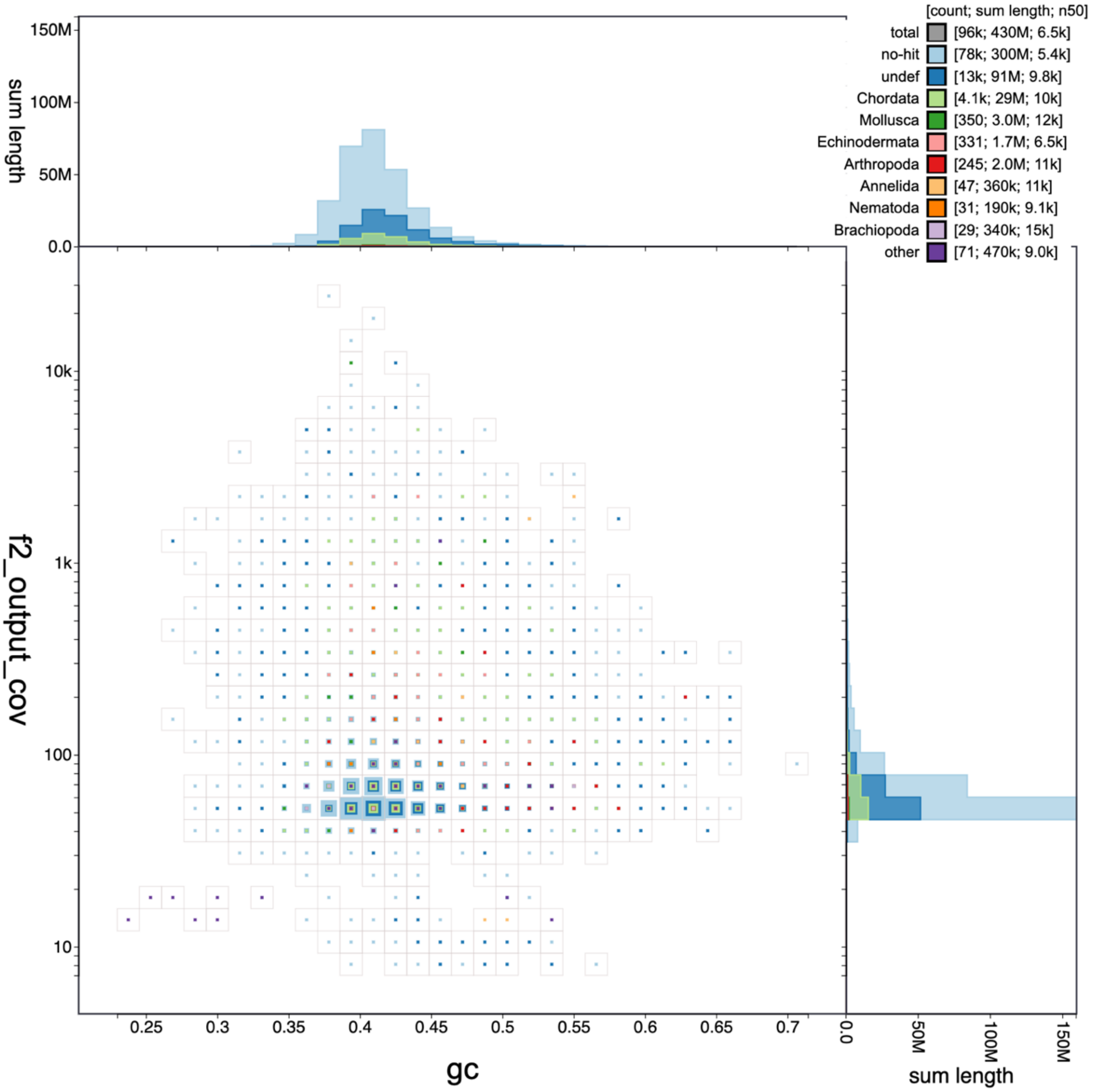

Figure S1. Square-binned blob plot of the *B. viridis* assembly displaying a single cluster.

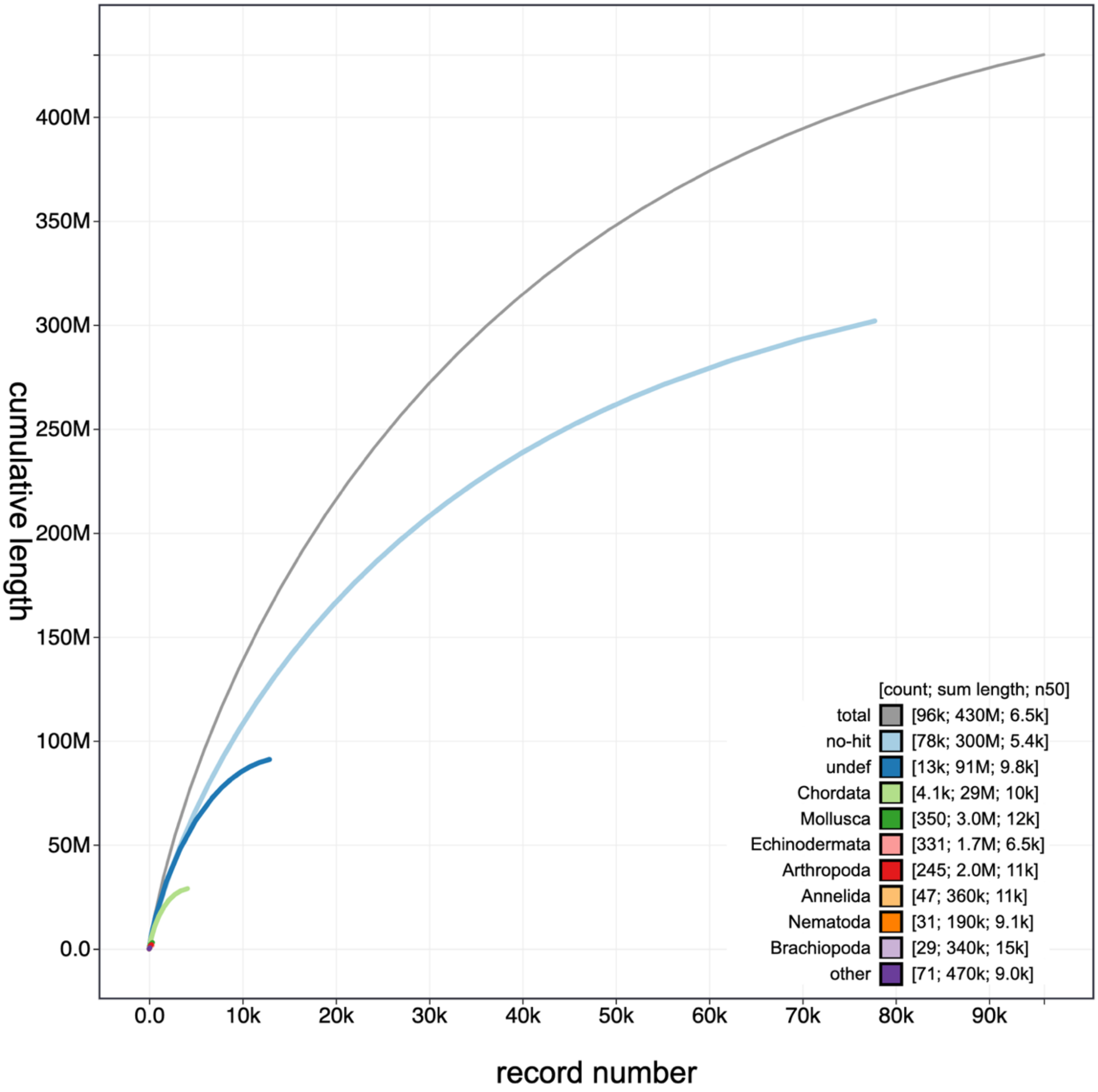

Figure S2. Cumulative assembly span plot of the *B. viridis* assembly displaying the assembly contiguity and record hits by taxon.

Transcriptome Re-assembly and Annotation

Paired-end Illumina sequence data publicly available on NCBI (SRA: SIO-Ec13/SRR2017645) were trimmed and assembled using SeqMan NGen (version 17.3, Lasergene, Madison, WI, USA) with a fragment size of 250 bp. Approximately 87.9 million raw 150 bp reads (43.8 million read pairs) were recovered (Table S4). Of these, 24.2 million assembled into 30,625 contigs (transcripts) with an average length of 648 bp, 4,843 of which were over 1 kb in length (Table S5).

Table S4. Sequence read summary statistics of the Illumina paired-end transcriptome assembly.

Total Assembled Reads	24230869
Total Unassembled Reads (saved)	16965182
Total Reads Excluded by Sampling (not saved)	46760917
Total Number of Reads	87956968

Table S5. Transcript summary statistics of the Illumina paired-end transcriptome assembly.

Total number of Transcripts	30625
Average Length of Assembled Transcripts	648
Assembled Transcripts >1kb	4843

BUSCO analysis was performed to assess the completeness of the *B. viridis* transcriptome assembly using the Eukaryota reference dataset (eukaryota_odb10). A total of 61.6% of near-universal single-copy orthologs were detected in the assembly, including 37.7% complete BUSCOs and 23.9% fragmented BUSCOs, while 38.4% were missing. Among the complete BUSCOs, 37.3% were present as single-copy genes and 0.4% were duplicated.

Furthermore, when utilizing the Gene Ontology (GO) database, the greatest number of sequences recovered at a node (7,203) were related to cellular processes, with the next greatest number of sequences (2,023) related to biological regulation (Figure S3).

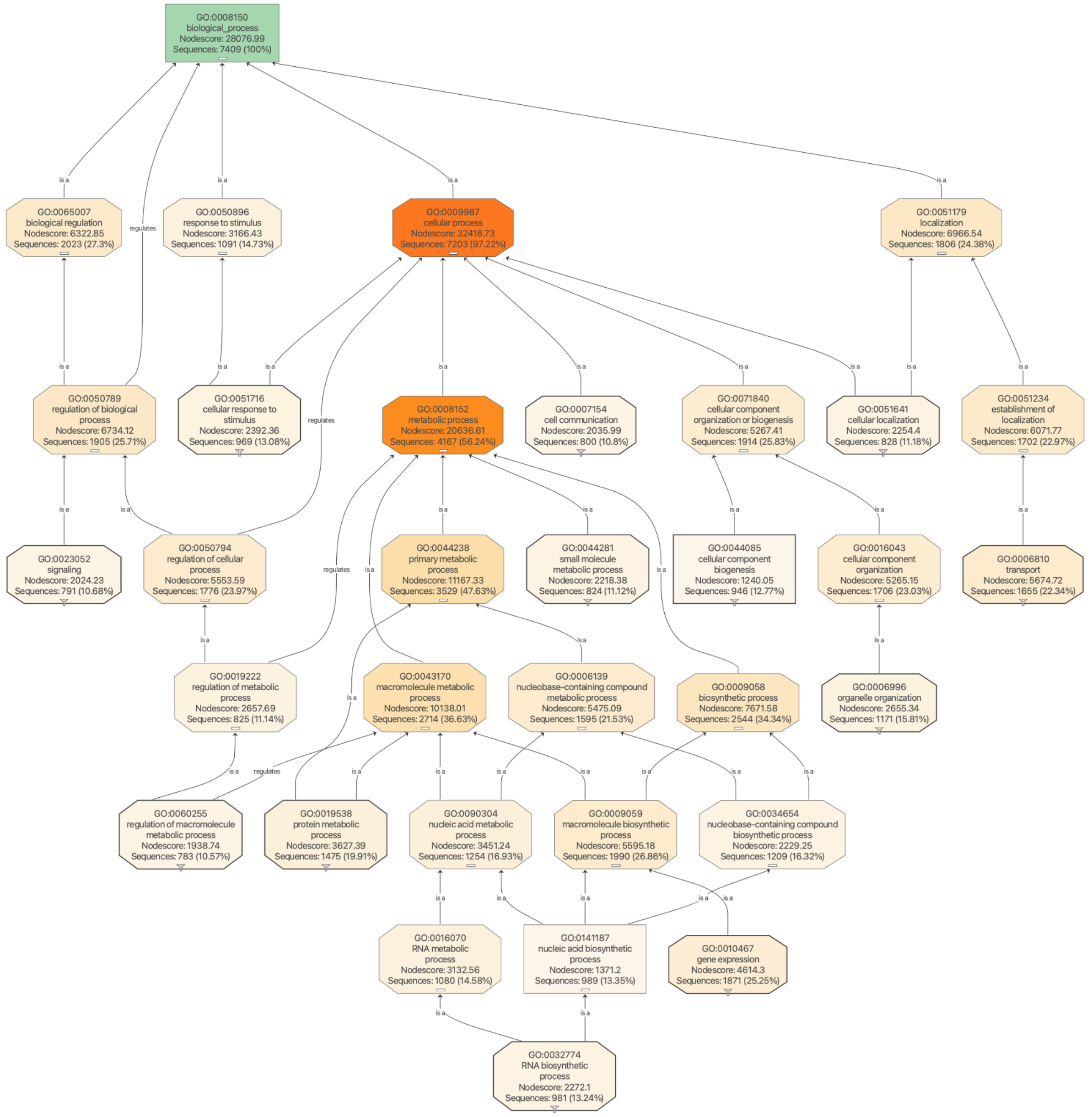

Figure S3. Map of functional transcript annotations from the *B. viridis* transcriptome (Illumina) re-assembly.
